# Higher risk of respiratory infections and otitis media in cleft lip and/or palate patients: the Japan Environment and Children’s Study

**DOI:** 10.1265/ehpm.24-00150

**Published:** 2024-11-27

**Authors:** Hiroshi Kurosaka, Takashi Kimura, Jia-Yi Dong, Meishan Cui, Satoyo Ikehara, Kimiko Ueda, Hiroyasu Iso, Takashi Yamashiro, the Japan Environment and Children’s Study Group

**Affiliations:** 1 Department of Orthodontics and Dentofacial Orthopedics, Graduate School of Dentistry, Osaka University, Japan; 2 Department of Public Health, Hokkaido University Graduate School of Medicine, Japan; 3 Public Health, Department of Social Medicine, Osaka University Graduate School of Medicine, Japan; 4 Osaka Maternal and Child Health Information Center, Osaka Women’s and Children’s Hospital, Japan

**Keywords:** Cleft lip and/or palate, Upper respiratory inflammation, Otitis media, Large cohort

## Abstract

**Background:**

Cleft lip and/or palate (CL/P) is one of the most frequent craniofacial disorder which could associate with a wide range of craniofacial complication. In order to perform comprehensive care of CL/P patients, it is crucial to elucidate the link of CL/P and general clinical conditions. This study aims to elucidate the relationships between medical history of different CL/P types and infectious diseases to serve as a reference for the comprehensive care of patients with CL/P.

**Methods:**

We investigated the association between a history different types of CL/P and the risk of infectious diseases among 1-year old children in the Japan Environment and Children’s Study (JECS). Among the 104,065 registered fetal records, 92,590 eligible participants were included in the analysis.

**Results:**

The multivariable-adjusted risk ratios (95% confidence intervals) for otitis media were increased in cleft lip and palate (CLP) and cleft palate only (CPO) groups by 3.81 (2.73–5.31) and 2.27 (1.22–4.22), respectively. The prevalence of Upper respiratory inflammation (URTI) was not associated with CLP, cleft lip only, or CPO. However, analysis in all groups showed a higher risk of URTI compared with the control group (1.31 [1.04–1.66]).

**Conclusions:**

CL/P care requires additional attention to prevent airway infectious diseases such as URTI before 1 year of age. Further research is warranted to elucidate the relationship between CL/P and general medical conditions.

**Supplementary information:**

The online version contains supplementary material available at https://doi.org/10.1265/ehpm.24-00150.

## Introduction

Orofacial cleft, which includes cleft lip and/or palate (CL/P), is one of the most common congenital disorders and leads to a variety of craniofacial malformations, such as midfacial deficiency [1]. Its etiology is multifactorial; various genetic and environmental factors are associated with the condition [2]. Embryonic cellular and molecular defects can result in CL/P; these common mechanistic problems influence the development of other tissues or organs, causing a wide range of congenital diseases [3–5]. Another important aspect to consider when providing comprehensive care to patients with CL/P are the possible sequelae. The general condition of patients with CL/P is influenced by disrupted craniofacial structures and the outcome of multiple surgeries, such as lip and palate closure. Craniofacial deformities and malocclusion due to maxillary growth retardation are well known issues in patients with CL/P [1]. Other oral functions such as speech and ingestion are also affected [6, 7]. However, knowledge of the relationship between CL/P and various general diseases remains limited. One of the most common general medical conditions in CL/P is otitis media, caused by inadequate eustachian tube ventilation [8]. A recent study showed that 1-year-old infants with orofacial clefts have a potentially high incidence of lower respiratory tract infections [9]. Otitis media and lower respiratory tract infections are inflammatory diseases; we hypothesized that patients with CL/P may be more sensitive to infections in general. Moreover, CL/P frequently results in physical connection between the oral and nasal cavities, which could be associated with complications in oral pharyngeal functions such as vocalization and swallowing [6, 7]. These features can also promote infection and inflammation of the respiratory tract. However, few studies have focused on the relationship between CL/P and risk of infectious diseases. Therefore, we investigated the association between the history of CL/P and risk of infectious diseases before 1 year of age in a large-scale cohort of Japan Environment and Children’s Study (JECS) [10].

## Methods

### Study cohort

The details of the JECS, a nationwide birth cohort study, including its protocol, have been described previously [11, 12].

Briefly, a self-administered questionnaire was distributed to all participants when they were registered during pregnancy. A second questionnaire survey was conducted during the second or third trimester of pregnancy, followed by surveys at 1 month and every 6 months after delivery. Information on infectious diseases occurring in infants till 1 year after delivery was collected using 6-month and 1-year questionnaires. Demographic information, including socioeconomic status, medical and obstetric history, physical and mental health, and lifestyle factors, was obtained. Written informed consent was obtained from all participants.

The JECS protocol was reviewed and approved by the Institutional Review Board on Epidemiological Studies of the Ministry of the Environment and the ethics committees of all participating institutions.

### Study population

**Fig. 1:**
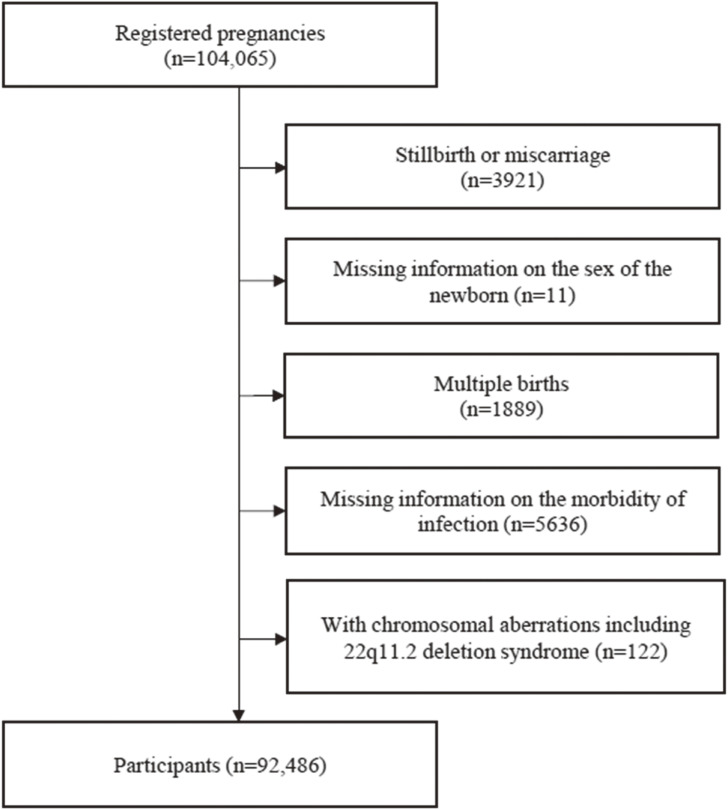
Flowchart of participant selection.

This study was based on the JECS-an-20180131 dataset released in March 2018. Among a total of 104,065 fetuses registered in the JECS study in 2014, we excluded stillbirths or miscarriages (n = 3,921), registrations lacking information on the newborn’s sex (n = 11), multiple births (n = 1,889), lack of information on the morbidity of infections (n = 5,636), and registrations with chromosomal aberrations including 22q11.2 deletion syndrome (n = 122). Finally, 92,486 fetuses were included in the analyses (Fig. 1).

### Orofacial cleft

The presence of cleft lip only (CLO), cleft lip and palate (CLP), and cleft palate only (CPO) was determined by a review of medical record transcripts at birth and 1 month after delivery. CL/P refers to all cases of cleft lip and/or palate.

### Main study outcome

Infectious disease was the main outcome of this study. Information about infectious diseases was obtained from self-administered questionnaires at 6 months and 1 year after delivery. At these timepoints, we collected information regarding the children’s history of otitis media, upper respiratory inflammation, and influenza. History of these diseases was assessed using the following question: “Has your child been diagnosed with a disease (by a doctor) after birth? (Check all that apply).” The infectious disease section included checkboxes for “Otitis media,” “Upper respiratory tract infection (including cold),” and “Flu.” We defined a check in one of these boxes in either questionnaire (6 months or 1 year) as the presence of infectious diseases until 1 year of age and a check in the box in the 6 months questionnaire as the presence of infectious diseases until 6 months of age.

### Confounding variables

The confounding variables were maternal age, study area, alcohol habits (never, former drinker, current drinker, or missing data), smoking habit (never, former smoker but quit before discovering current pregnancy, former smoker but quit after discovering current pregnancy, current smoker, or missing data), daily folate intake, folate supplements (yes, no, or missing data), educational level of the mother (junior high school, high school, career college or vocational school, university or graduate school, or missing data), breast feeding (Breastfeeding until 6 months of age (exclusively or mixed feeding), switched to formula milk at some point, or missing data) and infant food use for 6 months after birth.

Information on alcohol consumption habits, smoking habits, daily folate intake, and folate supplement use was collected from questionnaires administered during the first trimester [13]. Information on the mother’s educational level was collected from questionnaires administered during the second or third trimester. Information of breast feeding was collected from questionnaires for 6 months of age.

### Statistical analyses

Numbers and proportions of participants’ demographic characteristics, including children’s nutritional intake and mothers’ lifestyle habits, socioeconomic status, and folate intake from food and supplements, were calculated separately for patients with and without an orofacial cleft. We also performed robust multivariable Poisson regression analyses to estimate the adjusted risk ratios (RRs) (95% confidence interval [CI]) according to the presence or absence of orofacial cleft, adjusting for potential confounding variables which are considered to influence the etiology of both CL/P and infectious diseases (smoking habit, alcohol intake, folate intake, folate supplement use before pregnancy, educational level, and use of infant food).

In Table 2, “Model a” was adjusted for maternal age and study area while “Model b” was adjusted further for maternal education level, folic acid intake and supplementation, alcohol intake, smoking status, and use of baby food.

The distribution of missing values was estimated using multiple imputation with 25 imputations, where the multiple imputation models included orofacial cleft and all the adjusted variables.

All statistical analyses were performed using SAS version 9.4 (SAS Institute Inc., Cary, NC, USA). All reported *p*-values were two-sided, and significance was set at *p* < 0.05.

## Results

Table 1 shows the participant characteristics based on the presence of an orofacial cleft. We observed a similar prevalence of current drinkers, smokers before pregnancy, university or higher education, and folate intake before pregnancy between the orofacial cleft and control groups. However, the ratio of breastfeeding at 6 months of age was lower in the CLP (20.4%) and CPO (29.6%) groups than in the control (72.2%) and CLO (63.5%) groups.

**Table 1:** Characteristics of participants according to with or without orofacial cleft.

	**Control group (reference)**	**CLP**	**CLO**	**CPO**
Maternal factors				
Age, mean (SD)	30.8 (5.0)	30.2 (5.4)	30.7 (4.7)	30.5 (4.7)
Educational levels, N (%)				
Junior high school	4028 (4.4)	3 (3.2)	2 (3.2)	N.A.
Highschool	28261 (30.6)	36 (38.7)	19 (30.2)	16 (36.4)
2-year college, vocational school, or technical college	38668 (41.9)	27 (29.0)	30 (47.6)	21 (47.7)
University	20087 (21.8)	24 (25.8)	10 (15.9)	7 (15.9)
Missing	1242 (1.4)	3 (3.2)	2 (3.2)	N.A.
Folate intake during pregnancy, %				
T1 (Low)	30559 (33.1)	29 (31.2)	16 (25.4)	16 (36.4)
T2	30560 (33.1)	29 (31.2)	18 (28.6)	14 (31.8)
T3 (High)	30547 (33.1)	33 (35.5)	27 (42.9)	14 (31.8)
Missing	620 (0.7)	2 (2.2)	2 (3.2)	N.A.
Folate supplyment intake during pregnancy, %				
Yes	82945 (89.9)	83 (89.3)	57 (90.5)	36 (81.8)
No	7595 (8.2)	8 (8.6)	2 (3.2)	7 (15.9)
Missing	1746 (1.9)	2 (2.2)	4 (6.4)	1 (2.3)
Smoking status during pregnancy, %				
Non	53759 (58.3)	50 (53.8)	33 (52.4)	26 (59.1)
Ex- before pregnancy	21614 (23.4)	21 (22.6)	18 (28.6)	11 (25.0)
Ex- after pregnancy	11688 (12.7)	17 (18.3)	6 (9.5)	6 (13.6)
Current	3978 (4.3)	3 (3.2)	4 (6.4)	1 (2.3)
Missing	1247 (1.4)	2 (2.2)	2 (3.2)	N.A.
Drinking status during pregnancy, %				
Non	31674 (34.3)	34 (36.6)	24 (38.1)	15 (34.1)
Ex- before pregnancy	50558 (54.8)	52 (55.9)	33 (52.4)	23 (52.3)
Ex- after pregnancy	1105 (1.2)	2 (2.2)	1 (1.6)	2 (4.6)
Current	8077 (8.8)	3 (3.2)	3 (4.8)	4 (9.1)
Missing	872 (0.9)	2 (2.2)	2 (3.2)	N.A.
Infant				
Breastfeeding, %				
Breastfeeding until 6 months of age (exclusively or mixed feeding)	66630 (72.2)	19 (20.4)	40 (63.5)	13 (29.6)
Switched to formula milk at some point	24468 (26.5)	74 (79.6)	22 (34.9)	29 (65.9)
Missing	1188 (1.3)	N.A.	1 (1.6)	2 (4.6)
Babyfood using, %				
Yes	15484 (16.8)	27 (29.0)	21 (33.3)	9 (20.5)
No	76802 (83.2)	66 (71.0)	42 (66.7)	35 (79.6)

Abbreviation: CLP, cleft lip and palate; CLO, cleft lip; CPO, cleft palate.

A significantly higher incidence of otitis media was observed in the CLP and CPO groups, with age-adjusted and multivariable-adjusted relative risks (RRs) of 3.81 (2.73–5.31) and 2.27 (1.22–4.22), respectively. In contrast, the CLO group did not show a significant difference with age-adjusted and multivariable-adjusted RRs of 1.40 (0.76–2.57) and 1.42 (0.74–2.73), respectively (Table 2). A similar tendency for otitis media was seen in the analysis using the samples at 6 months of age (Supplementary Table 1).

**Table 2:** Prevalence ratios (95% CIs) of infections up to 1 year of age according to the presence or absence of orofacial cleft.

	**Control group (reference)**	**CLP**	**CLO**	**CPO**	**CL/P**
Otitis media					
No. of cases / No. at risk	10538 / 92286	35 / 93	9 / 63	10 / 44	54 / 200
RR (95% CI)a	1.00	3.68 (2.80–4.82)	1.40 (0.76–2.57)	2.27 (1.33–3.87)	2.65 (2.11–3.35)
RR (95% CI)b	1.00	3.81 (2.73–5.31)	1.42 (0.74–2.73)	2.27 (1.22–4.22)	2.71 (2.07–3.54)
Upper respiratory inflammation+Influenza					
No. of cases / No. at risk	29203 / 92286	33 / 93	23 / 63	20 / 44	76 / 200
RR (95% CI)a	1.00	1.14 (0.87–1.49)	1.17 (0.85–1.62)	1.45 (1.04–2.01)	1.22 (1.02–1.45)
RR (95% CI)b	1.00	1.19 (0.85–1.68)	1.20 (0.80–1.81)	1.44 (0.93–2.24)	1.25 (1.00–1.57)
Upper respiratory inflammation					
No. of cases / No. at risk	25800 / 92286	31 / 93	21 / 63	18 / 44	70 / 200
RR (95% CI)a	1.00	1.21 (0.91–1.61)	1.21 (0.86–1.71)	1.48 (1.03–2.11)	1.27 (1.05–1.53)
RR (95% CI)b	1.00	1.28 (0.90–1.81)	1.25 (0.81–1.91)	1.47 (0.92–2.33)	1.31 (1.04–1.66)
Influenza					
No. of cases / No. at risk	5051 / 92286	3 / 93	4 / 63	2 / 44	9 / 200
RR (95% CI)a	1.00	0.60 (0.20–1.84)	1.17 (0.45–3.03)	0.84 (0.22–3.26)	0.83 (0.44–1.58)
RR (95% CI)b	1.00	0.62 (0.20–1.92)	1.16 (0.44–3.09)	0.85 (0.21–3.41)	0.85 (0.44–1.63)

Abbreviation: CLP, cleft lip and palate; CLO, cleft lip only; CPO, cleft palate only; CL/P, cleft lip and/or palate.

a Adjusted for maternal age and study area.

b Adjusted further for maternal education level, folic acid intake, folic acid supplementation, alcohol intake, smoking status, breast feeding and baby food using.

We also observed a significantly higher incidence of upper respiratory inflammation in the CL/P group (all types), with age-adjusted and multivariable-adjusted RRs of 1.27 (1.05–1.53) and 1.31 (1.04–1.66), respectively. However, we observed no significant differences between the CL/P and control groups in the prevalence of influenza with a multivariable-adjusted RR of 0.85 (0.44–1.63).

## Discussion

The results of this large-scale nationwide study revealed a significant association between orofacial cleft and the risk of multiple infectious diseases, such as otitis media and URTI at 1 year of age. In addition to complications in the oral region, patients with CL/P frequently develop otitis media because of inadequate eustachian tube ventilation [8]. A cross-sectional study reported that >90% of ears in 55 patients with CLP and CPO exhibited otitis media at 20 months of age, compared with a cumulative incidence of approximately 50% in the general population before 3 years of age reported in a population-based birth cohort study that included 50,474 children with unknown genetic backgrounds [14, 15]. The present study detected a lower rate of otitis media in both the orofacial cleft and control groups than previously reported because the duration of sample collection was limited to 1 year after delivery. We observed a high RR of otitis media at 1 year of age in infants with CLP and CPO. In contrast, in the CLO group, this did not significantly differ from that in the control group. Several studies have investigated the risk of otitis media with different types of orofacial clefts, finding a higher risk in patients with CLP and CPO than in those with CLO, suggesting that a wider orofacial cleft could increase the risk of ear infection [16, 17]. Conversely, a case-control study of White participants <18 years old, which included 94 probands with CLO and 183 unaffected controls, reported a higher incidence of ear infection in CLO patients than in controls, with a multivariable OR (95%CI) of 3.70 (1.91–7.14) [18]. These controversial results for the incidence of otitis media in patients with CLO could be due to the different duration of sample collection. Altogether, this suggests that any type of orofacial cleft will potentially increase the risk of otitis media in the long run, a cleft in the secondary palate would increase this risk in infants even before 1 year of age. This large-scale prospective cohort study aimed to report the risk of otitis media in patients with CL/P in Japan. Given previous reports of racial diversity in the incidence of hearing loss in cleft palate patients, continuous investigation of the relationship between CL/P and otitis media in specific races is required [19, 20].

We also observed a high incidence of URTI in patients with CL/P. Another result from the JECS showed a higher incidence of lower respiratory tract infections in infants with CLP and CLO, with an adjusted incidence risk ratio (95%CI) of 2.38 (1.30–4.36) and 2.73 (1.40–5.33), respectively [9]. One possible reason for the high incidence of respiratory inflammation is the physical connection between the nasal and oral cavities, which may result in an environment susceptible to infection. There is also a possibility that URTI and otitis media can occur as a continuum and that CL/P patients have higher chance of developing otitis media resulting from URTI. A previous population-based case-control study of 1,203 cases of obstructive sleep apnea and 6,015 controls aged ≤18 years showed a strong association between an orofacial cleft and obstructive sleep apnea (adjusted OR [95%CI] 39.7 [16.8–93.9]) [21]. Further, a cross-sectional study reported that a higher incidence of respiratory infections was associated with sleep disorders (multivariable OR [95%CI] 1.88 [1.39–2.53]) [22]. These results indicate that in patients with CL/P who exhibit sleep disorders, the incidence of URTI or otitis media could be reduced by improving the immune reaction; this also needs to be studied in the future. Therefore, comprehensive follow-up with a pediatrician to monitor sleep status should be considered in patients with CL/P.

Although patients with CL/P may have an increased sensitivity to certain viral infections, highly contagious viruses, such as influenza, cause widespread outbreaks, leading to similar infection rates between participants with and without CL/P. Additionally, the risk of having either URTI or influenza showed the same tendency as the risk of having URTI alone, which again indicates limited risk of influenza for CL/P patients compared with the control group at one year of age.

The strength of this study was the large sample size and prospective data, which enabled us to analyze the association between CL/P history and risk of several infectious diseases. This study had several limitations. First, we could not include the severity of orofacial cleft, such as laterality of the cleft lip (bilateral or unilateral), or the extent to which the cleft existed (complete cleft or partial cleft). Cases with different cleft severities and types exhibit different clinical results, such as craniofacial growth and nasal airway dimensions, which could influence the sensitivity of patients to respiratory infections [23, 24]. Future studies are required to include a detailed classification and severity of orofacial clefts to better understand the mechanism of infection in CL/P patients [25, 26]. Second, the outcomes of infection were reported by mothers, which could introduce subjective bias. In particular, it is challenging to clinically distinguish between URTI and influenza, which could influence the results by over- or underestimating the number of URTIs. Third, at 1 year of age, some patients may have already undergone lip-and-palate closure under general anesthesia which is associated with complications such as respiratory infections [27]. Fourth, although the associations were adjusted for possible confounding factors, unmeasured confounding factors may have existed. Especially, factors such as posture during feeding and gastroesophageal reflux which are shown to increase the risk of otitis media could not be considered in this study because of lack of questionnaire [28, 29].

In conclusion, patients with CL/P showed a higher incidence of certain infectious diseases, presumably related to craniofacial deformities. Our results indicate the need for increased medical attention for certain infectious diseases in patients with CL/P.

## Supplementary information

**Additional file 1:** **Supplementary table 1.** Prevalence ratios (95% CIs) of infections up to 6 months of age according to the presence or absence of orofacial cleft.

## Declarations

### Availability of data and materials

The datasets generated and/or analysed during the current study are not publicly available due to ethical restrictions and legal framework of Japan. It is prohibited by the Act on the Protection of Personal Information (Act No. 57 of 30 May 2003, amendment on 9 September 2015) to publicly deposit the data containing personal information. Ethical Guidelines for Medical and Health Research Involving Human Subjects enforced by the Japan Ministry of Education, Culture, Sports, Science and Technology and the Ministry of Health, Labour and Welfare also restricts the open sharing of the epidemiologic data. All inquiries about access to data should be sent to: jecs-en@nies.go.jp. The person responsible for handling enquiries sent to this e-mail address is Dr Shoji F. Nakayama, JECS Programme Office, National Institute for Environmental Studies.
