# Supplementary material for: Higher risk of respiratory infections and otitis media in cleft lip and/or palate patients: the Japan Environment and Children’s Study
Source: Environ Health Prev Med. 2024 Nov 27;29:66. doi: 10.1265/ehpm.24-00150 (PMC11605134; doi:10.1265/ehpm.24-00150)
Supplement: Supplementary file 1 — Additional file 1: Supplementary table 1. Prevalence ratios (95% CIs) of infections up to 6 months of age according to the presence or absence of orofacial cleft. [file ehpm-29-066-s001.pdf]

Supplementary table1. Prevalence ratios (95% CIs) of infections up to 6 months of age according to the presence or absence of orofacial cleft.

	Control group (reference)	CLP	CLO	CPO	CL/P
Otitis media					
No. of cases / No. at risk	2179 / 92286	12 / 93	1 / 63	2 / 44	15 / 200
RR (95% CI)[a]	1.00	6.30 (3.70-10.74)	0.77 (0.11-5.49)	2.30 (0.62-8.60)	3.68 (2.26-6.00)
RR (95% CI)[b]	1.00	6.05 (3.43-10.68)	0.70 (0.10-4.97)	2.27 (0.57-9.07)	3.49 (2.10-5.81)
Upper respiratory inflammation+Influenza					
No. of cases / No. at risk	13666 / 92286	16 / 93	11 / 63	7 / 44	34 / 200
RR (95% CI)[a]	1.00	1.19 (0.76-1.85)	1.19 (0.69-2.05)	1.10 (0.55-2.20)	1.17 (0.86-1.59)
RR (95% CI)[b]	1.00	1.22 (0.74-1.99)	1.17 (0.65-2.12)	1.08 (0.52-2.27)	1.17 (0.84-1.64)
Upper respiratory inflammation					
No. of cases / No. at risk	12599 / 92286	15 / 93	11 / 63	6 / 44	32 / 200
RR (95% CI)[a]	1.00	1.21 (0.76-1.92)	1.30 (0.75-2.23)	1.02 (0.48-2.18)	1.19 (0.87-1.65)
RR (95% CI)[b]	1.00	1.24 (0.75-2.06)	1.28 (0.71-2.31)	1.01 (0.45-2.24)	1.20 (0.85-1.70)
Influenza					
No. of cases / No. at risk	1255 / 92286	1 / 93	1 / 63	1 / 44	3 / 200
RR (95% CI)[a]	1.00	0.80 (0.11-5.65)	1.17 (0.17-8.18)	1.67 (0.24-11.65)	1.11 (0.36-3.42)
RR (95% CI)[b]	1.00	0.78 (0.11-5.55)	1.10 (0.15-7.82)	1.64 (0.23-11.68)	1.07 (0.35-3.33)

Abbreviation: CLP, cleft lip and palate; CLO, cleft lip only; CPO, cleft palate only; CL/P, cleft lip and/or palate.

a Adjusted for maternal age and study area.

b Adjusted further for maternal education level, folic acid intake, folic acid supplementation, alcohol intake, smoking status, breast feeding and use of baby food
